# Supplementary material for: Mass Spectrometry-Based Quantification of Orexin Species in Human Cerebrospinal Fluid Reveals Differential Dynamics Associated with Sleep
Source: medRxiv. 2025 Dec 22:2025.12.06.25341754. Preprint. [Version 3] doi: 10.64898/2025.12.06.25341754 (PMC12704621; doi:10.64898/2025.12.06.25341754)
Supplement: Supplement 1 [file media-1.pdf]

Supplemental Table 1: Mixed Model Results for Longitudinal Orexin Peptides				
**Absolute Concentration, pg/ml (23:00-11:00)**				
OXA (RIA)				
Factor	Pairwise Comparison	Mean Difference	F (df)	p-value
Intervention	SD vs. C	+26.3 pg/ml	6.9 (1,32)	**0.013**
Time			1.3 (5,32)	0.297
Intervention x Time			1.9 (5,32)	0.119
Prepro-Orexin (MS)				
Factor	Pairwise Comparison	Mean Difference	F (df)	p-value
Intervention	SD vs. C	+59.4 pg/ml	8.5 (1,32)	**0.006**
Time			0.84 (5,32)	0.534
Intervention x Time			0.16 (5,32)	0.975
Long OXA with Intact N-Terminal (MS)				
Factor	Pairwise Comparison	Mean Difference	F (df)	p-value
Intervention	SD vs. C	+16.2 pg/ml	24.3 (1,32)	**<0.001**
Time			3.01 (5,32)	**0.024**
Intervention x Time			0.104 (5,32)	0.991
Short OXA with Intact N-Terminal (MS)				
Factor	Pairwise Comparison	Mean Difference	F (df)	p-value
Intervention	SD vs. C	+135.2 pg/ml	16.97 (1,32)	**<0.001**
Time			2.2 (5,32)	0.078
Intervention x Time			2.1 (5,32)	0.093
Long OXB with Truncated N-Terminal (MS)				
Factor	Pairwise Comparison	Mean Difference	F (df)	p-value
Intervention	SD vs. C	+52.8 pg/ml	23.9 (1,32)	**<0.001**
Time			1.3 (5,32)	0.281
Intervention x Time			0.29 (5,32)	0.918
Short OXB with Truncated N-Terminal (MS)				
Factor	Pairwise Comparison	Mean Difference	F (df)	p-value
Intervention	SD vs. C	+20.6 pg/ml	26.5 (1,32)	**<0.001**
Time			1.5 (5,32)	0.235
Intervention x Time			0.21 (5,32)	0.957
OXA: orexin-A; OXB: orexin-B; SD: sleep deprived; C: control; MS: mass spectrometry; RIA: radioimmunoassay; pg: picogram; ml: milliliter				

Supplemental Table 2: Mixed Model Results for Longitudinal Orexin Peptides				
**Percent of the Mean (23:00-11:00)**				
OXA (RIA)				
Factor	Pairwise Comparison	Mean Difference	F (df)	p-value
Intervention	SD vs. C	+2.7%	1.3 (1,32)	0.264
Time			1.9 (5,32)	0.126
Intervention x Time			2.5 (5,32)	**0.048**
Prepro-Orexin (MS)				
Factor	Pairwise Comparison	Mean Difference	F (df)	p-value
Intervention	SD vs. C	+9.6%	4.5 (1,32)	**0.042**
Time			0.72 (5,32)	0.616
Intervention x Time			0.4 (5,32)	0.842
Long OXA with Intact N-Terminal (MS)				
Factor	Pairwise Comparison	Mean Difference	F (df)	p-value
Intervention	SD vs. C	+25.8%	17.7 (1,32)	**<0.001**
Time			5.2 (5,32)	**0.001**
Intervention x Time			0.22 (5,32)	0.952
Short OXA with Intact N-Terminal (MS)				
Factor	Pairwise Comparison	Mean Difference	F (df)	p-value
Intervention	SD vs. C	+12.9%	3.7 (1,32.7)	0.063
Time			2.03 (5,32.6)	0.101
Intervention x Time			1.9 (5, 32.6)	0.126
Long OXB with Truncated N-Terminal (MS)				
Factor	Pairwise Comparison	Mean Difference	F (df)	p-value
Intervention	SD vs. C	+14.7%	7.4 (1,32)	**0.011**
Time			2.7 (5,32)	**0.040**
Intervention x Time			0.997 (5,32)	0.435
Short OXB with Truncated N-Terminal (MS)				
Factor	Pairwise Comparison	Mean Difference	F (df)	p-value
Intervention	SD vs. C	+16.01%	15.2 (1,32)	**<0.001**
Time			2.98 (5,32)	**0.025**
Intervention x Time			0.611 (5,32)	0.692
OXA: orexin-A; OXB: orexin-B; SD: sleep deprived; C: control; MS: mass spectrometry; RIA: radioimmunoassay				

**Supplemental Table 3: Mixed Model Results for Longitudinal Orexin Peptides**				
**Orexin Stable Isotope Labeling Kinetics (07:00-19:00)**				
Prepro-Orexin (Normalized TTR%)				
Factor	Pairwise Comparison	Mean Difference	F (df)	p-value
Intervention	SD vs. C	+0.102%	0.414 (1,14)	0.531
Time			71.66 (3,14)	**<0.001**
Intervention x Time			2.602 (3,14)	0.093
Long OXA with Intact N-Terminal (Normalized TTR%)				
Factor	Pairwise Comparison	Mean Difference	F (df)	p-value
Intervention	SD vs. C	+0.004%	0.862 (1,14)	0.369
Time			2.537 (3,14)	0.099
Intervention x Time			0.296 (3,14)	0.828
Long OXB with Truncated N-Terminal (Normalized TTR%)				
Factor	Pairwise Comparison	Mean Difference	F (df)	p-value
Intervention	SD vs. C	+0.014%	8.174 (1,14)	**0.013**
Time			19.05 (3,14)	**<0.001**
Intervention x Time			0.876 (3,14)	0.477
**Newly-Generated Orexin Peptides (07:00-19:00)**				
Newly-Generated Prepro-Orexin (MS)				
Factor	Pairwise Comparison	Mean Difference	F (df)	p-value
Intervention	SD vs. C	+2.073 pg/ml	1.97 (1,14)	0.182
Time			32.94 (3,14)	**<0.001**
Intervention x Time			1.042 (3,14)	0.404
Newly-Generated Long OXA with Intact N-Terminal (MS)				
Factor	Pairwise Comparison	Mean Difference	F (df)	p-value
Intervention	SD vs. C	+0.066 pg/ml	2.176 (1,14)	0.162
Time			2.819 (3,14)	0.077
Intervention x Time			0.293 (3,14)	0.830
Newly-Generated Long OXB with Truncated N-Terminal (MS)				
Factor	Pairwise Comparison	Mean Difference	F (df)	p-value
Intervention	SD vs. C	+0.967 pg/ml	34.61 (1,14)	**<0.001**
Time			20.62 (3,14)	**<0.001**
Intervention x Time			2.786 (3,14)	0.080
OXA: orexin-A; OXB: orexin-B; SD: sleep deprived; C: control; MS: mass spectrometry; TTR: tracer-tracee ratio; pg: picogram; ml: milliliter				

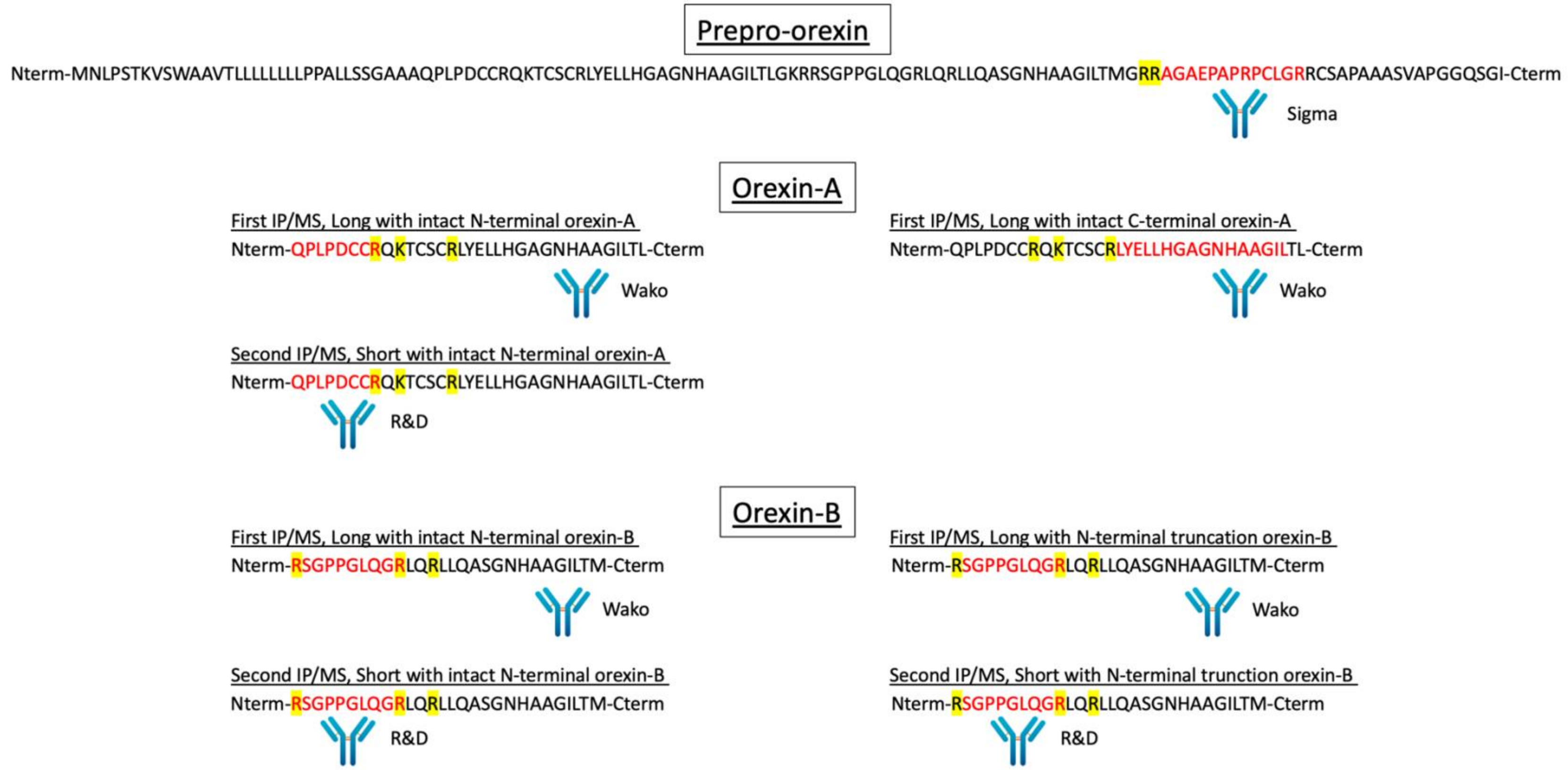

Supplemental Figure 1: Prepro-orexin, orexin-A, and orexin-B peptides measured by mass spectrometry. Yellow: Trypsin cleavage sites. Red: peptides measured by mass spectrometry.

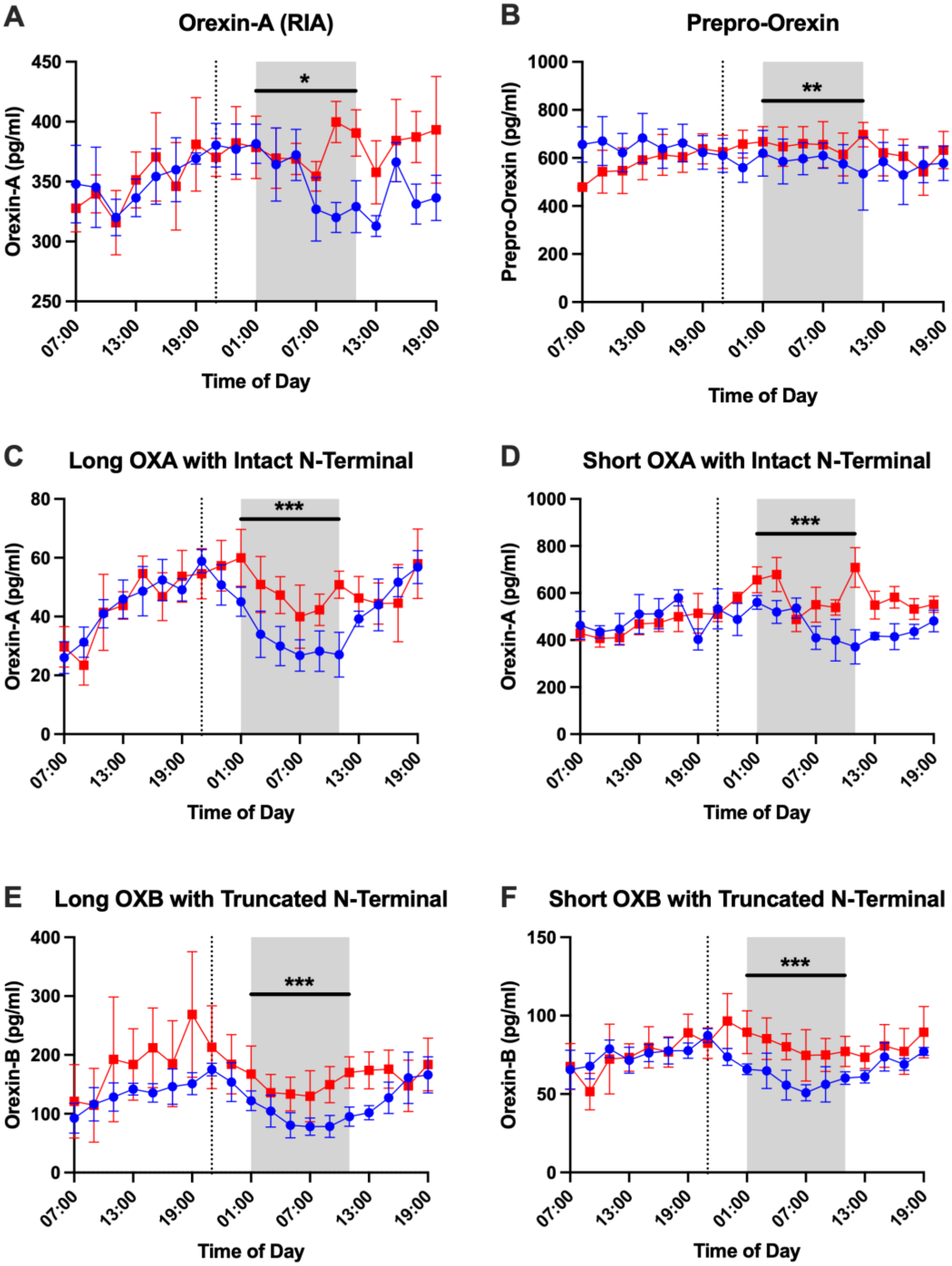

Supplemental Figure 2: Effect of overnight sleep deprivation on orexin peptide concentrations in cerebrospinal fluid. Four participants completed both the control normal sleep and sleep-deprived intervention groups. All orexin peptides are shown in pg/ml concentration. The overnight period during the intervention night was defined as hours 18 to 28 (01:00-11:00) to account for transit time of cerebrospinal fluid (CSF) from the brain to the lumbar catheter (shaded area). Blue: control; Red: sleep-deprived. Error bars indicate standard error. The vertical dashed line is the intervention start time. OXA: orexin-A. OXB: orexin-B. *p<0.05; **p<0.01; ***p<0.001.

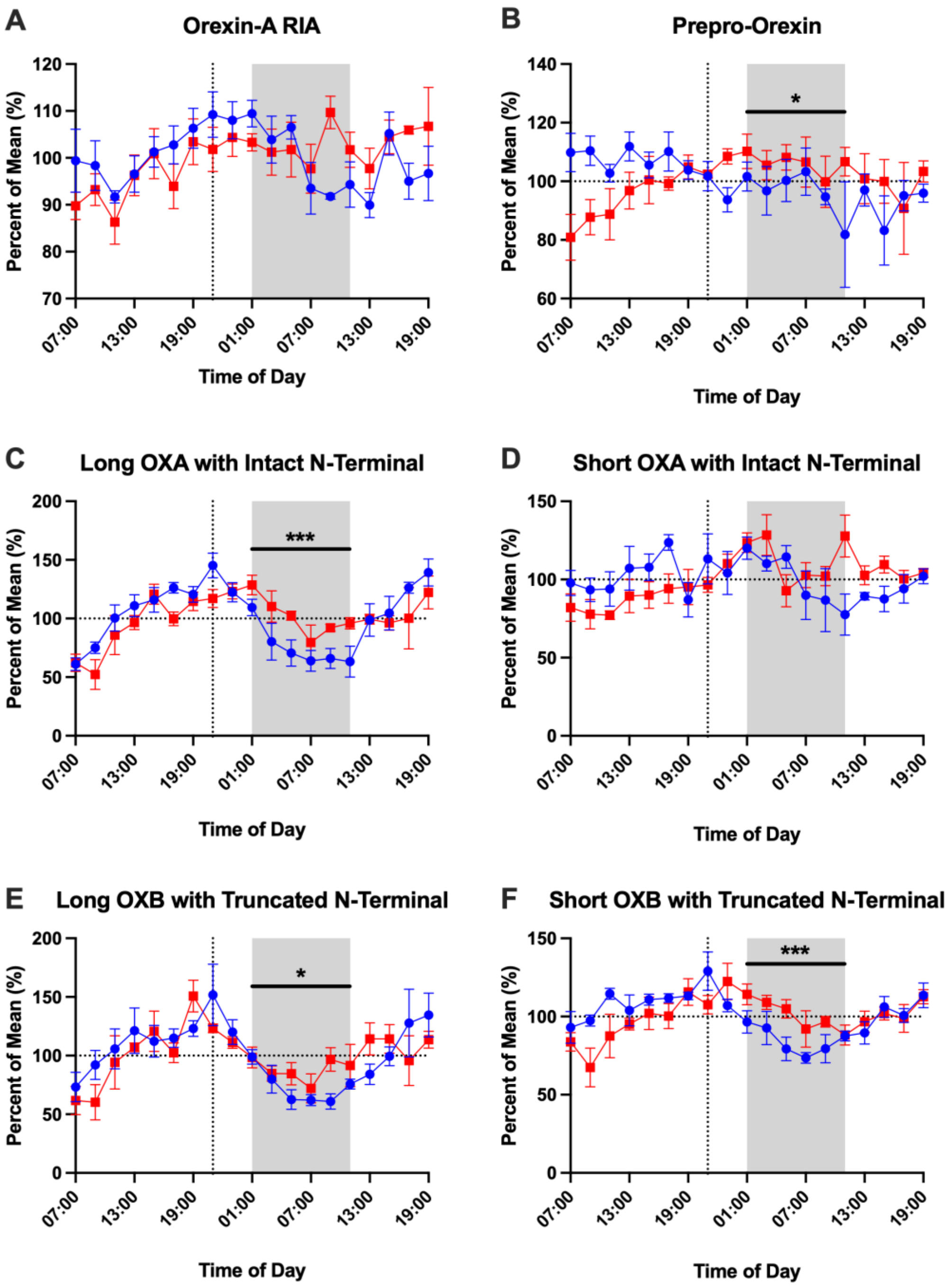

Supplemental Figure 3: Effect of overnight sleep deprivation on orexin peptide concentrations in cerebrospinal fluid normalized to the percent of the mean. Four participants completed both the control normal sleep and sleep-deprived intervention groups. All orexin peptides are shown as percent of the mean of each individual time course. Normalizing to the mean allows for comparing the diurnal oscillation between peptides on the same scale. The overnight period during the intervention night was defined as hours 18 to 28 (01:00-11:00) to account for transit time of cerebrospinal fluid (CSF) from the brain to the lumbar catheter (shaded area). Blue: control; Red: sleep-deprived. Error bars indicate standard error. The vertical dashed line is the intervention start time. OXA: orexin-A. OXB: orexin-B. *p<0.05; ***p<0.001
